# Activation of transsulfuration pathway by salvianolic acid a treatment: a homocysteine-lowering approach with beneficial effects on redox homeostasis in high-fat diet-induced hyperlipidemic rats

**DOI:** 10.1186/1743-7075-10-68

**Published:** 2013-12-06

**Authors:** Wenting Zhang, Hua He, Haidong Wang, Shijun Wang, Xi Li, Yao Liu, Huiyong Jiang, Hao Jiang, Yidan Yan, Yixuan Wang, Xiaoquan Liu

**Affiliations:** 1 Center for Drug Metabolism and Pharmacokinetics, China Pharmaceutical University, Tongjiaxiang 24, Gulou district, Nanjing 210009, Jiangsu, China

**Keywords:** Hyperlipidemia, Transsulfuration pathway, Homocysteine, Redox status, Salvianolic acid A

## Abstract

**Background:**

Elevated homocysteine is a cardiovascular risk factor in hyperlipidemia. Transsulfuration pathway provides an endogenous pathway for homocysteine conversion to antioxidant glutathione (GSH). Salvianolic acid A (Sal A) contains two molecules of caffeic acid and one molecule of danshensu that is capable of enhancing homocysteine transsulfuration, which led to the hypothesis that Sal A has activatory effect on transsulfuration pathway and this effect may have beneficial effects on both homocysteine and redox status in hyperlipidemia.

**Methods and results:**

To test this hypothesis, we developed a rat model of hyperlipidemia induced by high-fat diet for 16 weeks, during which rats were treated with 1 mg/kg salvianolic acid A (Sal A) for the final 4 weeks. Activities of key enzymes and metabolite profiling in the transsulfuration pathway revealed that hyperlipidemia led to elevated plasma homocysteine levels after 16-week dietary treatment, which was associated with reduced activities of homocysteine transsulfuration enzymes, cystathionine β-synthase (CBS) and cystathionine γ-lyase (CSE). The impaired transsulfuration pathway prevented homocysteine transsulfuration to cysteine, resulting in cysteine deficiency and subsequent reduction in GSH pool size. The redox status was altered in the setting of hyperlipidemia as indicated by GSH/GSSG ratio. Sal A treatment increased hepatic CBS and CSE activities, which was associated with reduced accumulation in circulating homocysteine levels and attenuated decline in hepatic cysteine content in hyperlipidemic rats. Sal A also led to an increase in GSH pool size, which subsequently caused a restored GSH/GSSG ratio. The activatory effect of Sal A on CBS was also observed in normal rats and in *in vitro* experiment.

**Conclusion:**

Our results suggest that activation of transsulfuration pathway by Sal A is a promising homocysteine-lowering approach that has beneficial effects on redox homeostasis in hyperlipidemic settings.

## Background

Hypercholesterolemia and hypertriglyceridemia are well-documented risk factors for the development of cardiovascular disease [1]. Homocysteine levels are elevated and considered as important indicators of atherosclerosis in hypercholesterolemia and hypertriglyceridemia [2].

**Figure 1:**
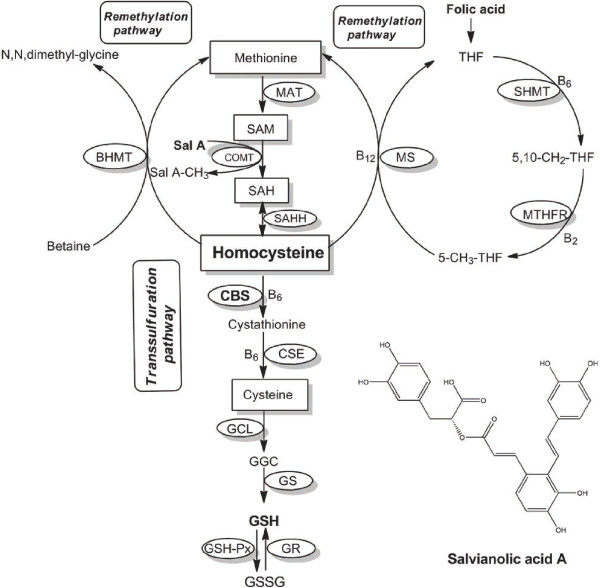
**Homocysteine metabolic pathways.** Abbreviations of the terms are as follows: MAT, methionine adenosyltransferase; COMT, catechol-O-methyltransferase; SAHH, S-adenosylhomocysteine hydrolase; BHMT, betaine-homocysteine methyltransferases; MS, methionine synthase; MTHFR, methylenetetrahydrofolate reductase; SHMT, serine hydroxymethyltransferase; CBS, cystathionine β-synthase, CSE, cystathionine γ-lyase; SAM, S-adenosylmethionine; SAH, S-adenosylhomocysteine; THF, tetrahydrofolate; GSH, reduced glutathione; GSSG, oxidized glutathione; GGC, γ-glutamylcysteine; GCL, glutamate-cysteine ligase; GS, GSH synthase; GSH-Px, glutathione peroxidase; GR, glutathione reductase.

Homocysteine is a thiol-containing amino acid, which suffers two major metabolic fates: remethylation catalyzed by methionine synthase (MS), methylenetetrahydrofolate reductase (MTHFR) or betaine-homocysteine methyltransferases (BHMT) and transsulfuration catalyzed by cystathionine β-synthase (CBS) leading to cystathionine [3] (Figure 1). Most of the current homocysteine-lowering therapies are based on the supplementation of folic acid that can facilitate the remethylation of homocysteine [4]. However, clinical trials showed that folic acid does not have beneficial effects on cardiovascular outcomes [5,6]. It is thus necessary to develop a non-folic acid homocysteine-lowering approach for hyperlipidemia, because the lack of benefit of homocysteine-lowering therapy suggests that folic acid-based treatment that is still most widely used may increase the cardiovascular risk that has already been elevated in the hyperlipidemic setting.

Besides remethylation, homocysteine can be eliminated through its transsulfuration [7]. Increasing evidence support the importance of the transsulfuration pathway in the maintenance of the redox homeostasis [3,8]. The transsulfuration pathway provides an endogenous pathway to utilize homocysteine for production of antioxidant glutathione (GSH). GSH, the most abundant intracellular nonprotein thiol, plays a key role in intracellular defense, and decreased concentrations of this antioxidant are correlated with the increased frequency of reactive oxygen species (ROS)-mediated mitochondrial damage and apoptosis [9,10]. GSSG is the oxidized form of glutathione, and the ratio between GSH and GSSG is fundamental for maintaining the redox status balance and important cellular functions, such as cell proliferation [11]. Redox imbalance can result in key events determining or associated with the onset and progression of the cardiovascular diseases, such as heart failure [12]. Therefore, transsulfuration pathway likely promotes the cardiovascular protection via redox regulation.

In the transsulfuration pathway, CBS is the first and rate-limiting enzyme for homocysteine conversion to cysteine, the availability of the latter controls the rate of GSH synthesis [13]. Retrospective studies have demonstrated that some beneficial polyphenols have the capacity to increase the activity of CBS [14,15]. Salvianolic acid A (Sal A), an aqueous extract from *salvia miltiorrhiza*, has been shown to present wide cardioprotective effects in myocardial ischemia, endothelial dysfunction and diabetes with most of the beneficial effects being related to its antioxidant capacity *in vivo*[16-18]. Previous investigations supported the most potent protective capacity of Sal A against oxidative damage among *salvia miltiorrhiza*-originated polyphenols including danshensu, salvianolic acid B, *et al.*, in liver microsomes, hepatocytes and erythrocytes of rats [19]. Sal A contains 1 molecule of danshensu (3,4-dihydroxyphenyllactic acid) and 2 molecules of caffeic acid (3,4-dihydroxycinnamic acid), the latter, a common coffee polyphenol, is the dehydration product of danshensu. Previous work in our laboratory has demonstrated that danshensu has potential to lower homocysteine levels via enhancing the transsulfuration pathway [20], leading to the possibility that part of the potent antioxidant effect of Sal A might be due to its activatory effect on transsulfuration pathway.

Along the lines mentioned above, we reasoned that Sal A might have activatory effect on transsulfuration pathway and this effect could show beneficial effects on homocysteine and redox homeostasis in the hyperlipidemic setting. To test the possibility, we developed a rat model with hyperlipidemia and treated the rats with Sal A. Concentrations of circulating homocysteine as well as its downstream products and key enzymes activities in the transsulfuration pathway were measured. The redox status was measured by determination of GSH/GSSG ratio. Our results indicate that Sal A indeed activates the transsulfuration pathway, suggesting a new approach to lower homocysteine with beneficial effects on redox homeostasis in hyperlipidemic settings.

## Methods and procedures

### Ethics statement

This study was carried out in strict accordance with the recommendations in the Guide for the Care and Use of Laboratory Animals of the National Institutes of Health. All experimental protocols were approved by Ethics Committee for Animal Experimentation of China Pharmaceutical University. All efforts were made to minimize animal suffering.

### Chemicals

Unless stated otherwise all chemicals used in these studies were obtained from Sigma Chemical (Shanghai, China). Sal A was supplied by Qingfeng Co. Ltd (Jiangxi, China).

### Animals, treatments, and tissue sampling

Male Sprague–Dawley rats (Super-B & K, Ltd., Shanghai, China) weighing 120–140 g were randomly divided into four groups with 6 rats per group. In the control group, normal rats were fed on basic diet prepared for 16 weeks and were given saline (ip) for the final 4 weeks during 16-week basic diet treatment; in the hyperlipidemia group, rats were fed on a high-fat diet composed of 56.9% basic diet, 13% lard, 2% sesame oil, 20% refined sugar, 3% cholesterol, 0.1% sodium cholate and 5% peanuts for 16 weeks and were given saline (ip) for the final 4 weeks during 16-week high-fat diet treatment; in the control + Sal A group, normal rats were fed on basic diet prepared for 16 weeks and were given Sal A (1 mg/kg/day, dissolved in saline, ip) for the final 4 weeks during 16-week basic diet treatment; in the hyperlipidemia + Sal A group, rats were fed on a high-fat diet for 16 weeks and were given Sal A (1 mg/kg/day, dissolved in saline, ip) for the final 4 weeks during 16-week high-fat diet treatment. All rats were allowed *ad libitum* access to food and water throughout the study. Finally, all these rats were sacrificed. The plasma was collected, tissues were rapidly removed, snap-frozen, and stored at −80°C until use. The rats were fasted for 6 hours before the plasma collection.

### Analytical procedures

The plasma homocysteine levels and hepatic cysteine, GSH, cysteinyl-glycine (Cys-Gly) concentrations were measured according to the previous method [21]. Rat livers were homogenized in PBS buffer (0.1 mol/L, pH 7.4) to prepare a 20% (wt/vol) homogenate for measurement. S-adenosylmethionine (SAM) and S-adenosylhomocysteine (SAH) in the liver was measured based on previous method [22]. Plasma cholesterol, triglycerides, LDL-cholesterol and hepatic GSSG concentrations were measured spectrophotometrically by commercial diagnostic kits (Jiancheng Institute of Biotechnology, Nanjing, China).

The protein concentration was quantified by bicinchoninic acid (BCA) assay [23]. The CBS activity assay was determined by cystathionine formation, as previously described with modification [24,25]. Briefly, rat livers were homogenized in PBS buffer (0.1 mol/L, pH 7.4) to prepare a 20% (wt/vol) homogenate. After centrifugation, liver protein supernatant was incubated with reaction mixture (0.1 mol/L serine, 5 mmol/L EDTA, and 2.5 mmol/L propargylglycine in Tris buffer (1 mol/L, pH 8.4)), 2.5 mmol/L pyridoxalphosphate and homocysteine reagent for 1 h at 37°C. The homocysteine reagent consisted of 0.03 g homocysteine in 1 mL of 2.5 mol/L KOH solution. This reagent was neutralized by addition of a mixture of 2.57 mL of 4.5 mol/L HCl and 4.43 mL of Tris buffer (1 mol/L, pH 8.4). The mixture was incubated at 37°C for 60 min and terminated by 50% (vol/vol) trichyperlipidemiaoroacetic acid, followed by the centrifugation at 10,000 g for 5 min. The supernatant was mixed with ninhydrin solution (0.8% (wt/vol) in glacial acetic acid). The mixture was boiled for 5 min and cooled on ice for 2 min. The absorbance of the solution was determined at 455 nm. The activities of glutamate-cysteine ligase (GCL) and glutathione synthase (GS) were measured together using the previous method [26]. The activity was determined from the rate of formation of GSH measured as described above. The activity of cystathionine γ-lyase (CSE) in the liver was determined by α-ketobutyrate generation as description of previous studies with modification [27]. Rat livers were homogenized in PBS buffer (0.1 mol/L, pH 7.4) to prepare a 20% (wt/vol) homogenate. After centrifugation, liver protein supernatant was incubated with reaction mixture containing 32 mmol/L homoserine, 0.1 mmol/L pyridoxalphosphate, 7.5 mmol/L 2-mercaptoethanol, 7.0 mmol/L EDTA and 0.1 mol/L PBS (pH 7.4). The incubation was carried at 37°C for 30 min, followed by adding 20% trichyperlipidemiaoroacetic acid (wt/vol). The amount of α-ketobutyrate, which was generated during the enzymatic reaction, was quantified with 3-methyl-2-benzothiazolinone hydrazine hydrochyperlipidemiaoride as the description of previous studies [28].

### *In vitro* effect of Sal A on CBS activity

To assess the effect of Sal A on CBS activity *in vitro*, Sal A was pre-incubated with normal rat liver homogenate (20%, w/v in PBS buffer (0.1 mol/L, pH 7.4)) as source of CBS activity at 37°C for 15 min, afterwards, CBS activity was determined as described previously. The Sal A concentrations in homogenate were 0, 0.1, 0.2, 0.5, 1, 2, 4, 10, 25, 50 μmol/L.

### Statistical analysis

All data were represented as the means ± SEM. Statistical analysis was calculated by a one-way ANOVA with Newman-Keuls test using GraphPad prism 5.0 (GraphPad Software, SanDiego, CA, USA). The relationship between two variables was analyzed by use of linear regression analysis. The acceptable level of significance was established at *p* < 0.05.

## Results

### Body weight and plasma lipid profile

Following 16 weeks of high-fat diet feeding, body weight was significantly greater in the hyperlipidemia group (+21% *vs.* control group, *p* < 0.05) (Table 1). In hyperlipidemia group, plasma cholesterol, triglycerides as well as LDL-cholesterol concentrations were significantly higher than in control group (*p* < 0.05). After Sal A treatment, in hyperlipidemic rats, no significant changes were observed in body weight, cholesterol and triglycerides levels in plasma. In contrast, hyperlipidemia-induced elevation of plasma LDL-cholesterol levels was reduced by Sal A treatment (−20% *vs.* hyperlipidemia group, *p* < 0.001).

**Table 1:** Body weight, plasma cholesterol, triglycerides and LDL-cholesterol concentrations

	**Control**	**Control + Sal A**	**Hyperlipidemia**	**Hyperlipidemia + Sal A**
Body weight (g)	351 ± 9	353 ± 7	425 ± 19^*^	423 ± 21
Cholesterol (mmol/ L)	1.52 ± 0.04	1.53 ± 0.01	2.92 ± 0.16^***^	2.64 ± 0.20
Triglycerides (mmol/ L)	1.06 ± 0.04	1.04 ± 0.11	1.50 ± 0.12^*^	1.37 ± 0.12
LDL-cholesterol (mmol/ L)	0.93 ± 0.03	1.03 ± 0.03	1.52 ± 0.09^***^	1.21 ± 0.05^###^

Data are represented as means ± SEM, n = 6.

^*^p < 0.05 (*vs.* control group). ^**^p < 0.01 (*vs.* control group). ^***^p < 0.001 (*vs.* control group).

^#^*p* < 0.05 (*vs.* hyperlipidemia group). ^##^*p* < 0.01 (*vs.* hyperlipidemia group). ^###^*p* < 0.001 (*vs.* hyperlipidemia group).

### Plasma homocysteine levels and activity profile of key enzymes for homocysteine transsulfuration in the liver

**Figure 2:**
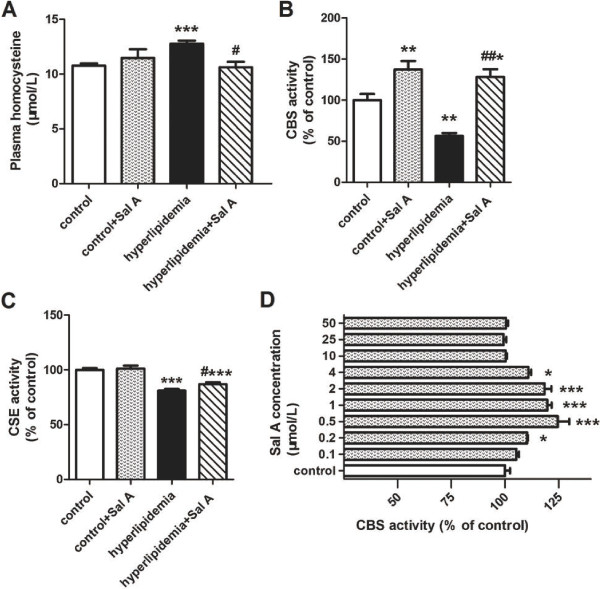
**Effect of Sal A on plasma homocysteine concentrations and hepatic homocysteine transsulfuration activity.** Plasma homocysteine levels **(A)**, and hepatic activities of homocysteine transsulfuration enzymes, CBS **(B)** and CSE **(C)**, in the normal rats (control) and hyperlipidemic rats after 4-week Sal A treatment (1 mg/kg/day, ip). Bars represent means ± SEM, n = 6. **(D) ***In vitro* effect of Sal A on CBS activity: liver homogenate as source of CBS activity was incubated with Sal A at the dose of 0.1, 0.2, 0.5, 1, 2, 4, 10, 25, 50 μmol/L at 37°C for 15 min. Control represents the CBS activity without Sal A. Bars represent means ± SEM, n = 8. ^*^p < 0.05 (*vs.* control group). ^**^p < 0.01 (*vs.* control group). ^***^p < 0.001 (*vs.* control group). ^#^*p* < 0.05 (*vs.* hyperlipidemia group). ^##^*p* < 0.01 (*vs.* hyperlipidemia group). ^###^*p* < 0.001 (*vs.* hyperlipidemia group).

**Figure 3:**
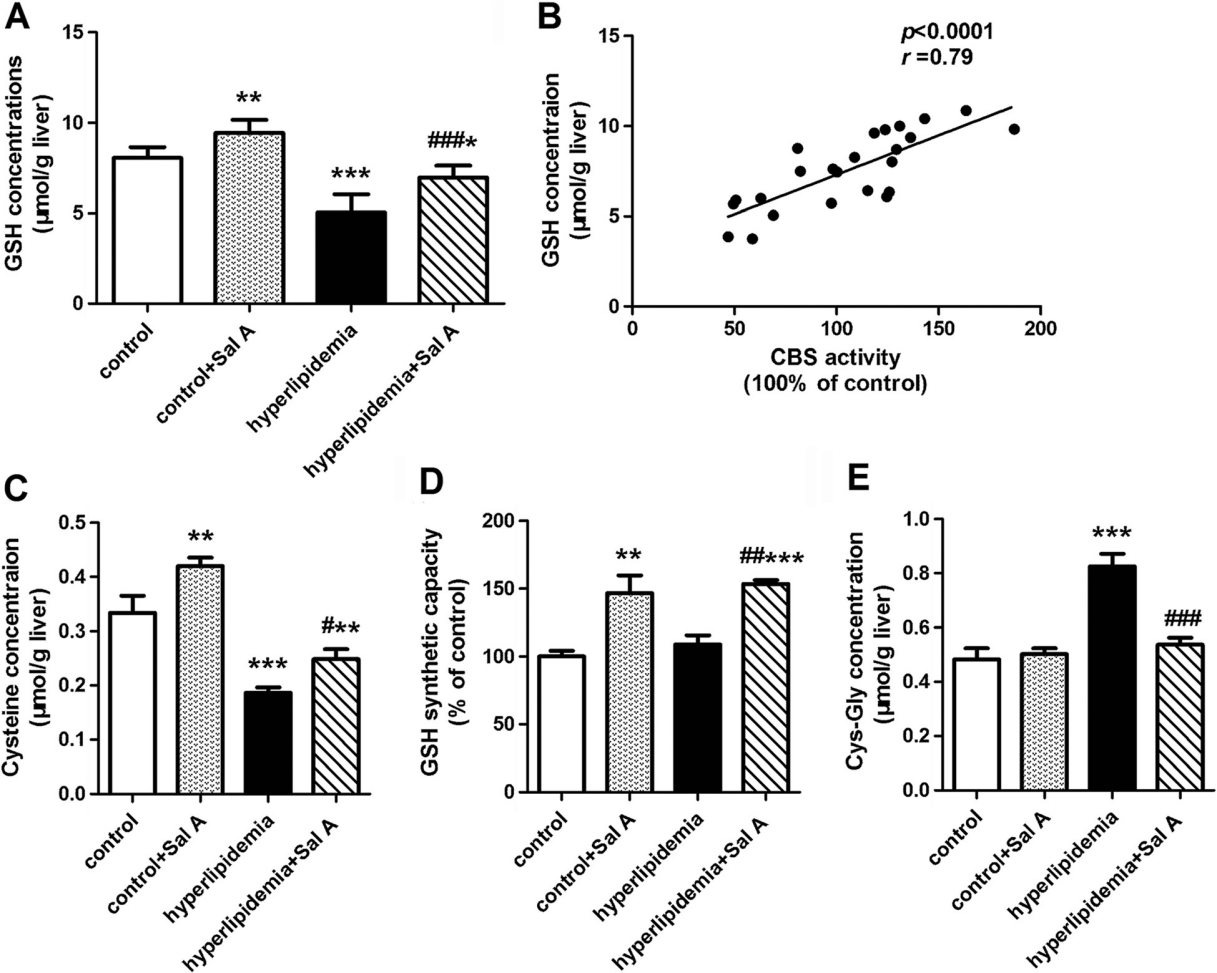
**Effects of Sal A on the concentrations of GSH and its relevant metabolites.** GSH concentrations **(A)** and its correlation with CBS activity **(B)** in the liver of normal rats (control) and hyperlipidemic rats after 4-week Sal A treatment (1 mg/kg/day, ip). Concentrations of GSH precursor cysteine **(C)**, GSH synthetic capacity **(D)** as well as concentrations of GSH extracellular metabolite Cys-Gly **(E)** in the liver of normal rats (control) and hyperlipidemic rats after 4-week Sal A treatment (1 mg/kg/day, ip). Bars represent means ± SEM, n = 6. ^*^p < 0.05 (*vs.* control group). ^**^p < 0.01 (*vs.* control group). ^***^p < 0.001 (*vs.* control group). ^#^*p* < 0.05 (*vs.* hyperlipidemia group). ^##^*p* < 0.01 (*vs.* hyperlipidemia group). ^###^*p* < 0.001 (*vs.* hyperlipidemia group).

Plasma homocysteine level was elevated in hyperlipidemic rats (12.78 ± 0.28 μmol/L) compared with control rats (10.78 ± 0.20 μmol/L, *p* < 0.001) (Figure 2A). The enzyme activity profiling for homocysteine transsulfuration in the liver where the lipid metabolism and homocysteine metabolism take place showed that hyperlipidemia led to a significant decrease in CBS activity (−44% *vs.* control group, *p* < 0.01) (Figure 2B) and CSE activity (−19% *vs.* control group, *p* < 0.001) (Figure 2C) in the liver, indicating impaired homocysteine transsulfuration to cysteine. Consistently, cysteine content was reduced in hyperlipidemia group (0.18 ± 0.01 μmol/g liver) compared with control group (0.33 ± 0.03 μmol/g liver, *p* < 0.001) (Figure 3C).

In hyperlipidemic rats, Sal A treatment caused enhanced activities of hepatic CBS (+2.2-fold *vs.* hyperlipidemia group, *p* < 0.01) and CSE (+7% *vs.* hyperlipidemia group, *p* < 0.05), which was accompanied by a reduction in plasma homocysteine levels (−17% *vs.* hyperlipidemia group, *p* < 0.05) and an increase in hepatic cysteine concentrations (+33% *vs.* hyperlipidemia group, *p* < 0.05).

In control rats, in response to Sal A treatment, hepatic activity of hepatic CBS was increased by 37% (*p* < 0.01) whereas CSE activity was unchanged compared with untreated normal rats. Meanwhile, hepatic cysteine content was increased by 26% (*p* < 0.01) whereas plasma homocysteine concentrations showed no statistically difference as compared with untreated controls.

Correlation analysis showed that hepatic CBS activity was positively correlated with hepatic cysteine concentrations (*r* = 0.59, *p* < 0.01, the curve was not shown).

### Sal A caused an increase in CBS activity in *in vitro* experiment

The Sal A increased CBS activity at the concentration of 0.2, 0.5, 1, 2, 4 μmol/L with significance (*p* < 0.05) (Figure 2D). The Sal A activated about 25% of the enzyme activity when incubated with liver homogenate for 15 min at 37°C at the concentration of 0.5 μmol/L (*p* < 0.001).

### Hepatic redox status

**Figure 4:**
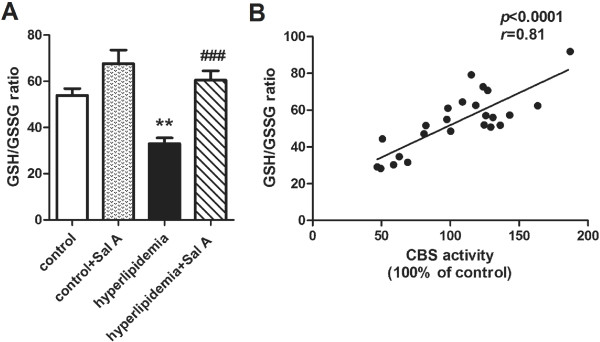
**Effect of Sal A on redox status.** Hepatic GSH/GSSG ratio **(A)** as well as its correlation with CBS activity **(B)** in the normal rats (control) and hyperlipidemic rats after 4-week Sal A treatment (1 mg/kg/day, ip). Bars represent means ± SEM, n = 6. ^*^p < 0.05 (*vs.* control group). ^**^p < 0.01 (*vs.* control group). ^***^p < 0.001 (*vs.* control group). ^#^*p* < 0.05 (*vs.* hyperlipidemia group). ^##^*p* < 0.01 (*vs.* hyperlipidemia group). ^###^*p* < 0.001 (*vs.* hyperlipidemia group).

The glutathione status (GSH/GSSG ratio), which accurately reflects intracellular redox status, was used as indicator of oxidative stress in this study [29]. A reduced GSH/GSSG ratio was observed in the liver of hyperlipidemic rats (−39% *vs.* control group, *p* < 0.01) (Figure 4A). Together with this, GSH concentration was also decreased (−37% *vs.* control group, *p* < 0.001) (Figure 3A) whereas GSSG concentrations were indistinguishable between control (0.15 ± 0.01 μmol/g liver) and hyperlipidemia group (0.17 ± 0.01 μmol/g liver). GSH synthesis depends on the availability of its rate-limiting precursor cysteine [30]. Deficiency of cysteine content (−44% *vs.* control group, *p* < 0.001) (Figure 3C) and unchanged GSH synthetic capacity (Figure 3D) indicated reduction of GSH production in the liver of hyperlipidemia group. Moreover, the hepatic concentration of Cys-Gly, an extracellular GSH metabolite, was elevated in hyperlipidemia group (+71% *vs.* control group, *p* < 0.001) (Figure 3E), which indicated an increased loss of GSH from the cell.

In response to Sal A treatment, cysteine content and enhanced GSH synthetic capacity were increased in both control (cysteine content: +26%, *p* < 0.01; GSH synthetic capacity: +46%, *p* < 0.01) and hyperlipidemia group (cysteine content: +33%, *p* < 0.05; GSH synthetic capacity: +41%, *p* < 0.01), which occurred with a decrease in the Cys-Gly concentration in the hyperlipidemia group (−35%, *p* < 0.001). This suggested enhanced GSH synthesis in both control and hyperlipidemia group and reduced GSH efflux from hepatocytes in the hyperlipidemia group after Sal A treatment. Consistently, GSH concentrations was elevated in the liver of both control (+17%, *p* < 0.01) and hyperlipidemia (+38%, *p* < 0.001) groups. Meanwhile, together with increased GSH levels, hyperlipidemia group showed a decline in the GSSG levels (−31%, *p* < 0.05) and an elevation in GSH/GSSG ratio (+83%, *p* < 0.001) after Sal A treatment, which indicated attenuated oxidative stress.

Correlation analysis showed that CBS activity was strongly and positively correlated with GSH concentrations (*r* = 0.79, *p* < 0.0001) (Figure 3B) and GSH/GSSG ratio (*r* = 0.81, *p* < 0.0001) (Figure 4B).

### Hepatic methylation status

A decrease in SAM/SAH ratio was predictive of reduced methylation capacity in hyperlipidemia group (−43% *vs.* control group, *p* < 0.01) (Table 2). After Sal A treatment, SAM/SAH ratio was unchanged in the hyperlipidemia group whereas this ratio was decreased in the control group (−29%, *p* < 0.01).

**Table 2:** The methylation status in the liver

	**Control**	**Control + Sal A**	**Hyperlipidemia**	**Hyperlipidemia + Sal A**
SAM (nmol/g liver)	112.8 ± 10.8	83.0 ± 4.8^**^	64.2 ± 5.0^***^	60.3 ± 5.5
SAH (nmol/g liver)	26.7 ± 1.7	27.8 ± 1.4	27.9 ± 1.5	23.3 ± 1.6
SAM/SAH	4.2 ± 0.4	3.0 ± 0.2^**^	2.4 ± 0.3^**^	2.6 ± 0.3

Data are represented as means ± SEM, n = 6.

^*^p < 0.05 (*vs.* control group). ^**^p < 0.01 (*vs.* control group). ^***^p < 0.001 (*vs.* control group).

^#^*p* < 0.05 (*vs.* hyperlipidemia group). ^##^*p* < 0.01 (*vs.* hyperlipidemia group). ^###^*p* < 0.001 (*vs.* hyperlipidemia group).

## Discussion

Elevated plasma homocysteine level is a risk factor of atherogenesis [31]. In the present study, we found that Sal A was capable to lower elevated plasma homocysteine levels in the hyperlipidemic setting. This effect, which was relevant to the enhanced homocysteine transsulfuration, was due to the stimulatory effect of Sal A on CBS activity, the latter was also responsible for the alleviation of the redox imbalance in hyperlipidemia.

### Hyperlipidemia and elevated plasma homocysteine levels as well as cysteine deficiency

In our experiment, plasma homocysteine was elevated after 16-week high-fat dietary treatment as compared with normal controls. Activity profiling of transsulfuration enzymes suggested that impaired transsulfuration pathway characterized by reduced activities of CBS and CSE brought about depressed conversion of homocysteine to cysteine along the transsulfuration pathway, consequently leading to upstream homocysteine accumulation and downstream cysteine deficiency in the hyperlipidemia. Our results were consistent with previous studies and were supported indirectly by the studies that focused on inborn CBS deficiency [32]. CBS deficiency led to markedly homocysteine accumulation and severely decreased levels of cystathionine and cysteine, which was due to impaired homocysteine removal through the transsulfuration pathway [32]. Our findings in a hyperlipidemia model were consistent with previous studies which reported that 18-week high-fat feeding could cause hyperhomocysteinemia accompanied by cysteine deficiency, led to the speculation that the transsulfuration pathway was affected [33]. Down-regulated activities of CBS and CSE of the pathway confirmed the speculation and therefore led to their conclusion that high-fat-induced down-regulation of hepatic transsulfuration activity caused plasma homocysteine elevation, which was likely to contribute to the increased risk of cardiovascular disease associated with the condition [33].

### Hyperlipidemia and enhanced oxidative stress

Oxidative stress was enhanced in the hyperlipidemic rats, which was indicated by reduced GSH pool size and GSH/GSSG ratio.

GSH plays a central role in the antioxidant defence of cells against oxidative stress [34]. The GSH intracellular concentration reflects a dynamic balance between the synthesis and consumption of GSH within the cell and loss through efflux [35]. In the setting of hyperlipidemia, conspicuous reduction in GSH content in the liver was observed, which was accompanied by the reduction in the concentration of cysteine-the rate limiting precursor of GSH, suggesting that reduced availability of cysteine led to impaired GSH production, and subsequent decreased GSH pool size. The hepatic concentration of Cys-Gly, an extracellular metabolite of GSH degradation catalyzed by γ-glutamyl transpeptidase [36], was increased in hyperlipidemic rats compared with normal ones, indicating an increased secretion or GSH loss from the hepatocytes, followed by GSH degradation. This was also responsible for GSH depletion in hyperlipidemia.

In our study, no significant difference in hepatic GSSG concentrations was found between hyperlipidemic and normal rats. Under oxidative stress conditions, ROS are reduced by GSH with concomitant formation of the GSSG [37]. However, GSSG did not accumulation in the setting of hyperlipidemia. To maintain the cellular redox balance, GSSG is rapidly and efficiently exported out of the cell by ATP-dependent transport proteins under conditions of oxidative stress [38]. This would provide an explanation for unchanged GSSG content in this study.

### The transsulfuration pathway-the link between redox imbalance and homocysteine elevation in the setting of hyperlipidemia

GSH synthesis is dependent on the availability of cysteine [30], the latter is the downstream product of homocysteine in its transsulfuration. Actually, the effect of transsulfuration pathway on GSH concentrations is more than its effect on GSH synthesis, but also the GSH efflux from the cell. The loss of GSH through the membrane may in turn depend on membrane oxidative stress, because oxidative stress causes lipid peroxidation of cell membrane, leading to altered membrane permeability and transport functions and consequently increased GSH efflux [36]. The decrease of antioxidant GSH synthesis could make contributions to oxidative stress that in turn led to increased GSH loss to the extracellular space. The involvement of transsulfuration regulated GSH generation and exportation strengthens the importance of the role that transsulfuration pathway played in the redox control.

Our data showed that hyperlipidemia-induced transsulfuration impairment blocked the homocysteine conversion to cysteine, which led to not only upstream accumulation of homocysteine, but also downstream deficiency of cysteine and subsequent reduction of antioxidant GSH synthesis. This would further reduce the hepatic GSH content by means of enhancing the efflux of GSH from the intercellular space, and then further aggravate the redox imbalance.

In the setting of hyperlipidemia, the pathological effect of transsulfuration pathway on redox status is not that simple, because in normal physiological settings, the flux of homocysteine to GSH through the transsulfuration pathway can be compensatively enhanced in response to the oxidative stress in order to maintain the oxidant/antioxidant balance whereas antioxidant treatment elicit the opposite effect on transsulfuration pathway [8]. However, in the setting of hyperlipidemia, GSH depletion caused by disturbed transsulfuration could lead to the oxidant/antioxidant imbalance on its own, and the impaired transsulfuration could cause the loss of the compensatory GSH synthesis in response to oxidative stress, which would lead to further oxidative stress.

Along the lines above, transsulfuration pathway is likely to be a potential target for homocysteine-lowering and redox-rebalancing approach in the hyperlipidemic setting.

### Homocysteine accumulation was reduced in response to Sal A treatment in hyperlipidemia–the activatory effect of Sal A on CBS activity

In the transsulfuration pathway, CBS catalyzes the initial and rate-limiting step for homocysteine transsulfuration pathway [39]. The CBS activatory capacity was previously found in catechin and wind polyphenols [14,15]. In this study, after Sal A treatment, elevated plasma homocysteine levels and reduced hepatic cysteine concentrations returned toward normal when the activities of CBS and CSE were increased in hyperlipidemic rats. In addition, the activatory effect of Sal A on the CBS activity was found not only in the hyperlipidemic rats but also in the liver homogenate *in vitro*, and in the normal rats. These results suggested that Sal A enhanced the homocysteine removal via its transsulfuration to cysteine in hyperlipidemic rats by means of increasing CBS activity.

However, normal rats showed unchanged homocysteine concentration in the plasma, even though the CBS was activated and cysteine concentration was elevated after the Sal A treatment. Like other polyphenols, Sal A can accept methyl groups from SAM and cause an elevation of homocysteine during the methionine-homocysteine cycle (Figure 1) [20]. It is likely that the amount of homocysteine generation from SAH and the amount of homocysteine conversion to cysteine via transsulfuration pathway was comparable, leading to unchanged homocysteine concentration in normal rats after Sal A treatment. In contrast, the Sal A methylation can be inhibited because of the diminished SAM and consequent hypomethylation in hyperlipidemic rats (Table 2). In this circumstance, it is even more significant for the elimination of homocysteine through transsulfuration pathway than that of increase caused by the methylation of Sal A.

In this study, we found that Sal A also had potential to lower homocysteine levels via enhancing the transsulfuration of homocysteine like danshensu [20]. Sal A is composed of danshensu and its structural analog caffeic acid, indicating that the activatory effect of Sal A on homocysteine transsulfuration is probably conferred by its danshensu-containing structure. In retrospective, the exact step of transsulfuration pathway at which danshensu acts has not been tested and our current study revealed that Sal A activated CBS, the key enzyme for homocysteine transsulfuration pathway, to achieve its homocysteine-lowering effect. In addition, previous studies focused upon the effect of danshensu on homocysteine were conducted in a rat model of hyperhomocysteinemia that was induced by prolonged methionine dosing [20]. In that pathological model, plasma homocysteine accumulation was mainly attributed to increased homocysteine production from methionine but not impaired homocysteine conversion to cysteine. The effects of danshensu on impaired transsulfuration are unclear. Our present study showed that Sal A was capable to reduce the homocysteine elevation caused by transsulfuration block in hyperlipidemia.

### Oxidative stress was ameliorated in response to Sal A in hyperlipidemia–an increase in concentrations of downstream products of homocysteine in the transsulfuration pathway

Sal A led to ameliorated oxidative stress that was reflected by elevations of GSH/GSSG ratio in the hyperlipidemic rats. This elevation was due to increased GSH content and decreased GSSG content after Sal A treatment. In Sal A treated normal controls, GSH concentration was also elevated whereas GSH/GSSG ratio tended to increase but not significantly. Moreover, the GSH concentration and its ratio with GSSG concentration were strongly, and positively, correlated with hepatic CBS activity, indicating that Sal A-induced GSH increase and subsequent alteration in GSH/GSSG ratio was associated with its activatory effect on CBS activity. Consistently, our data showed that together with the increase of GSH content, cysteine concentrations as well as the CBS and GSH synthetic activities, were also increased in both hyperlipidemic and normal rats whereas the Cys-Gly concentrations were declined in hyperlipidemic rats after Sal A treatment. It was suggested that Sal A enhanced GSH synthesis due to its activatory effect on CBS and consequent homocysteine conversion to cysteine, which subsequently enhanced antioxidant defence and prevented redox-induced GSH loss, leading to further increase in GSH pool size and GSH/GSSG ratio in hyperlipidemic rats. This beneficial effects of Sal A on redox homeostasis was consistent with previous studies showing that Sal A exerted its protective effects against oxidative stress via reducing ROS accumulation in both animal and hepatocyte culture models [40,41]. The effect of Sal A on transsulfuration pathway and this pathway-dependent GSH synthesis found in this study provides a new sight into the antioxidant effect of Sal A besides its catechol structures and is suggested to be one of the multiple mechanisms accounting for the potential beneficial effects of Sal A.

### Limitations

In this study, we have demonstrated that Sal A has activatory capacity on homocysteine transsulfuration to antioxidant GSH. Despite the well documented relationship between oxidative stress and cardiovascular diseases [42], whether the Sal A treatment would eventually improve cardiovascular outcomes in the setting of hyperlipidemia is unknown, although the wide cardioprotective effects of Sal A have been studied and confirmed previously in myocardial infarction, and other pathological settings [16,43]. The possibility for Sal A to be used as a cardioprotective homocysteine-lowering therapy in hyperlipidemia needs to be further investigated.

## Conclusions

In conclusion, the present study supports the involvement of impaired transsulfuration pathway in the hyperlipidemia-induced homocysteine accumulation and redox imbalance. Furthermore, our data demonstrates that Sal A has activatory capacity on CBS activity and this effect makes it a promising homocysteine-lowering approach with beneficial effects on redox status in the setting of hyperlipidemia.

## Abbreviations

CBS: Cystathionine β-synthase; CSE: Cystathionine γ-lyase; Sal A: Salvianolic acid A; SAM: S-adenosylmethionine; SAH: S-adenosylhomocysteine; GSH: Reduced glutathione; GSSG: Oxidized glutathione; Cys-Gly: Cysteinyl-glycine; GCL: Glutamate-cysteine ligase; LDL: Low density lipoprotein; MS: Methionine synthase; BHMT: Betaine homocysteine methyltransferases; MTHFR: Methylenetetrahydrofolate reductase; ROS: Reactive oxygen species.

## Competing interests

The authors declare that they have no competing interests.

## Authors’ contributions

The contribution of each author to the present paper was as follows: WT Zhang, XQ Liu and H He were responsible for the research design; HD Wang, SJ Wang, WT Zhang, X Li, Y Liu, HY Jiang, H Jiang, YD Yan, YX Wang conducted the research; WT Zhang performed data analysis and statistical analysis; WT Zhang, XQ Liu and H He prepared the manuscript. All co-authors read and approved the final manuscript.
